# An Innovative Method for Rapid Identification and Detection of *Vibrio alginolyticus* in Different Infection Models

**DOI:** 10.3389/fmicb.2016.00651

**Published:** 2016-05-06

**Authors:** Kaifei Fu, Jun Li, Yuxiao Wang, Jianfei Liu, He Yan, Lei Shi, Lijun Zhou

**Affiliations:** ^1^Central Laboratory, Navy General HospitalBeijing, China; ^2^Medical Administrative Department, Navy General HospitalBeijing, China; ^3^Department of Biochemistry and Molecular Biology, Medical College, Qingdao UniversityQingdao, China; ^4^College of Light Industry and Food Sciences, South China University of TechnologyGuangzhou, China; ^5^Institute of Food Safety and Nutrition, Jinan UniversityGuangzhou, China

**Keywords:** *Vibrio alginolyticus*, *toxR* gene, real-time LAMP, 16s rDNA sequencing, rapid detection

## Abstract

*Vibrio alginolyticus* is one of the most common pathogenic marine *Vibrio* species, and has been found to cause serious seafood-poisoning or fatal extra-intestinal infections in humans, such as necrotizing soft-tissue infections, bacteremia, septic shock, and multiple organ failures. Delayed accurate diagnosis and treatment of most *Vibrio* infections usually result to high mortality rates. The objective of this study was to establish a rapid diagnostic method to detect and identify the presence of *V. alginolyticus* in different samples, so as to facilitate timely treatment. The widely employed conventional methods for detection of *V. alginolyticus* include biochemical identification and a variety of PCR methods. The former is of low specificity and time-consuming (2–3 days), while the latter has improved accuracy and processing time. Despite such advancements, these methods are still complicated, time-consuming, expensive, require expertise and advanced laboratory systems, and are not optimal for field use. With the goal of providing a simple and efficient way to detect *V. alginolyticus*, we established a rapid diagnostic method based on loop-mediated Isothermal amplification (LAMP) technology that is feasible to use in both experimental and field environments. Three primer pairs targeting the *toxR* gene of *V. alginolyticus* were designed, and amplification was carried out in an ESE tube scanner and Real-Time PCR device. We successfully identified 93 *V. alginolyticus* strains from a total of 105 different bacterial isolates and confirmed their identity by 16s rDNA sequencing. We also applied this method on infected mouse blood and contaminated scallop samples, and accurate results were both easily and rapidly (20–60 min) obtained. Therefore, the RT-LAMP assay we developed can be conveniently used to detect the presence of *V. alginolyticus* in different samples. Furthermore, this method will also fulfill the gap for real-time screening of *V. alginolyticus* infections especially while on field.

## Highlights

1. A new LAMP method was developed for the detection of *Vibrio alginolyticus*.
2. It successfully and accurately detected 105 *Vibrio* strains from different sources.
3. The assay was directly applicable to infected mouse blood and contaminated scallops.
4. The assay was suitable for use both in field and routine laboratories.

## Introduction

*Vibrio* species are pathogenic bacteria that are implicated in a number of serious infections and illnesses (Vezzulli et al., 2013). One of the most pathogenic species is *Vibrio alginolyticus*, a gram-negative and curved halophilic bacterium, initially classified as a biological type of *Vibrio parahaemolyticus*, but was later on listed as an independent species in Buchanan and Gibbons (1974). *V. alginolyticus* is mainly found in marine estuaries, coastal and aquatic environments (Narracci et al., 2013) with worldwide distribution. It may exist as free-living, a parasite or associated with surfaces of organisms such as marine vertebrates/invertebrates and flora, and even humans (Molitoris et al., 1985; Chen et al., 2011; Schets et al., 2011; Torresi et al., 2011). This species is also highly abundant and usually dominates *Vibrio* communities (Lai et al., 2004; Schets et al., 2010; Jones et al., 2013).

*Vibrio alginolyticus* has been found to cause varied infections and inflammation in both humans and animals (Rubin and Tilton, 1975) such as otitis, ocular infections, intracranial infection, peritonitis and osteomyelitis among others (Taylor et al., 1981; Opal and Saxon, 1986; Barbarossa et al., 2002; Feingold and Kumar, 2004; Li et al., 2009). In addition, serious extra-intestinal or seafood borne primary infections, especially in immunocompromised patients, can cause fatal diseases such as necrotizing soft-tissue infections and bacteremia, which are always accompanied by septic shock and multiple organ dysfunction, resulting in high morbidity and mortality (Chien et al., 2002; Hong et al., 2014). The number of cases associated with *V. alginolyticus* has dramatically increased in the past decades, posing threats to public health and safety, and even food security. In Chinese southeast coastal areas for example, cases of *V. alginolyticus*-associated wound infections and gastro-intestinal diseases are already considered not uncommon (Ji, 1989; Song et al., 2013). The same is true in the United States and other regions of the world (Weis et al., 2011; Jones et al., 2013). In fact, *V. alginolyticus* has been observed to cause infections leading to various illnesses in *Penaeus vannamei Boone*, *Epinephelus coioidesi*, *Crassostrea rhizophorae*, *Pseudosciaena crocea*, and *Sparus latus* among others (Pereira et al., 2007; Oates et al., 2012).

The severity and life-threatening characteristics of *V. alginolyticus* related infections necessitate a rapid, low-cost and accurate method of early and specific identification and detection of the pathogen, which would be helpful in saving lives. At present, the widely employed conventional methods include PCR amplification and identification of 16s rRNA gene, and biochemical characterizations (Lai et al., 2014). However, these traditional techniques are still limited with lower accuracy, longer processing time and cost of personnel and reagents.

Loop-mediated isothermal amplification (LAMP) is an innovative isothermal nucleic acid amplification technology that has been applied to gene diagnosis (Notomi et al., 2015). Compared with most known gene diagnostics (i.e., PCR, hybridization), LAMP has low requirement for complex infrastructure with easily readable results without compromising specificity and sensitivity. For example, LAMP has shortened the sample pre-preparation and detection time from 4 to 10 days (collection-extraction-PCR) to only 20–60 min. Because of such apparent advantages, LAMP has been given increased attention by the World Health Organization (WHO), relevant government departments, institutes and researchers all over the world. To date, the method has been successfully used for the rapid diagnosis of microorganisms such as viruses, bacteria and parasites in different samples (Cai et al., 2010; Liew et al., 2014; Ferrara et al., 2015; Hayashida et al., 2015; Liu et al., 2015; Wang et al., 2016).

A crucial parameter in the application of LAMP technology is the design of primers targeting genes of high specificity between species. Among the possible target genes used for the specific *Vibrio* detection, the concatenated sequences of *toxR* show a considerable gap between the maximal interspecies similarity and the minimal intraspecies similarity (Pascual et al., 2010). For instance, the similarity between *Chromobacterium violaceum*, *V. parahaemolyticus*, and *Vibrio vulnificus* is only 52–63% (Lin et al., 1993; Lee et al., 2000; Chen et al., 2012). This hypervariable characteristic of *toxR* gene makes it a good molecular marker for species identification. The *toxR* gene has been widely used in the identification of *Vibrio* species, such as *V. parahaemolyticus*, *V. anguillarum*, *V. vulnificus*, *Vibrio cholera* (Neogi et al., 2010; Crisafi et al., 2011; He et al., 2014). In this study, we aimed to establish a new rapid diagnostic LAMP method to identify the presence of *V. alginolyticus* by targeting the *toxR* gene.

## Materials and Methods

### Bacterial Strains and Growth Condition

A total of 105 marine bacterial strains were used in our study. Ninety-two marine strains were isolated from southeast China coastal waters by Navy General Hospital microbiological experiment. The other six marine bacteria were cultivated from seafood samples and were provided by Beijing Entry-Exit Inspection and Quarantine Bureau. Model and standard strains including *V. fluvialis* 1A10009, *V. parahaemolyticus* 1A10122, *V. fischeri* 1H00021, and *V. vulnificus* 1H00066 were purchased from MCCC (Marine Culture Collection of China), while *V. alginolyticus* 17749 was an ATCC standard saved by our laboratory. *Escherichia coli* MG4 was from Ocean University of China and *C. violaceum* CV026 was provided by the PLA Academy of Military Sciences.

All the bacteria in this study were pre-identified by physiological and biochemical characterization, and further purified by isolating single colonies. Cultures were routinely maintained and rec-culturing overnight in broth medium (2216E or LB broth) at 30 or 35°C.

### Optimization and Validation of Real-Time Lamp Assay

#### Lamp Primer Design Targeted toxR Gene

Loop-mediated Isothermal amplification primers targeting the *toxR* gene of *V. alginolyticus* were designed based on the sequence: GenBank = JF930593.1, using LAMP designer 1.13 (Premier Biosoft, USA). Designed primers and their sequences are summarized in **Table 1**. The primers were tested in all 105 strains to test for efficiency and specificity.

**Table 1:** Summary of primer sequences used for the RT-LAMP assay targeting the *toxR* gene.

Primer name^∗^	Sequence (5′–3′)
F3	AGGTAGTGACCGATACTAC
B3	ATGCATTGCTCTATTGAGG
FIP	ACGCGGTAGCCAGTTAATCTGATCTTGAGCCTCTAGTAG
BIP	GTCTCTGCTGCTTCCTGTTGATACTCACCAATCTGACG
FLP	TGTTAGATGCTGGCTTCG
BLP	GCGTATTGTTATTCACGAACC


*^∗^F3, forward outer primer; B3, reverse outer primer; FIP, forward inner primer; BIP, reverse inner primer; FLP, forward loop primer; BLP, reverse loop primer.*

### DNA Extraction

Two kinds of commercially available DNA extraction kits (BEIJING TIANGEN Biotech, Co., Ltd.) were adopted. TIANamp Bacteria DNA Kit was used for DNA extraction of bacteria grown in the culture medium. Target bacteria were adjusted to 1 × 10^6^ CFU/mL in advance. TIANamp Blood DNA Kit was used for whole blood DNA extraction, and 500 μL blood was needed of each sample. DNA was extracted from both types of samples according to the manufacturer’s protocols. All extracted DNA templates were stored at -20° for later use.

At the meantime, positive and negative controls were set. Plasmid DNA containing the target gene isolated from *V. alginolyticus* 17749 was used as the positive control for PCR. It was amplified using primers F3 and B3 (**Table 1**), and then linked to a T vector. Sterile ddH_2_O was used as the negative control.

#### Real-Time LAMP Reaction

Loop-mediated Isothermal amplification assays were carried out using the *Bst* WarmStart^TM^ DNA polymerase (New England Biolabs; 1 μL) in mixtures added with 22 μL buffer solution containing 0.2 μM F3, 0.2 μM B3, 1.6 μM FIP, 1.6 μM BIP, 0.8 μM FLP, 0.8 μM BIP, 1 M betaine, 8 mM MgSO_4_, 1.6 mM dNTP, 2.5 μL 10× Thermo pol buffer (New England Biolabs), 5 μM SYTO^®^-9 (Invitrogen, Life Technology) and ddH_2_O per reaction. Two microliters of DNA template were used per assay, totaling to 25 μL.

Isothermal amplification was accomplished at 63°C for 30 min while using ESE-Quant tube scanner as one of the two detection platforms, and the whole reaction system was sealed by addition of 200 μL mineral oil (GBCBIO Technologies). The other platform tested measures the amplification as it happens via a Real-Time PCR device (Bio-Rad Laboratories). Reaction started with 45 cycles at 63°C for 15 s (stage 1) and at 63°C for 45 s (stage 2) coupled with detection of fluorescence signals, which were analyzed by Bio-Rad CFX Manager 3.1. Through the two reaction monitoring systems, a result was considered positive when a “S” amplification curve was obtained, while linear or slightly oblique amplification curve showed a negative result.

#### Validation by 16s rDNA Sequencing

To validate the results of the LAMP assay, identities of the 105 bacterial strains were confirmed by 16s rDNA identification. Amplification of the target gene was done in 30 μL PCR reaction mixtures following the suggestion of Lane (1991) and Moreno et al. (2002). Primers used for 16s rDNA identification are shown in **Table 2**.

**Table 2:** Sequences of universal primers for 16s rDNA sequencing.

Target	Primer^∗^	Sequences from 5′ to 3′
16s rDNA	27F	AGAGTTTGATCCTGGCTCAG
	1492R	GGTTACCTTGTTACGACTT
	n27F	AGAGTTTGATCMTGGCTCAG
	n1492R	TACGGYTACCTTGTTACGACTT


*^∗^27F the forward primer and 1492R the reverse primer were for Vibrio bacteria; n27F the forward primer and n1492R the reverse primer were for non-Vibrio bacteria.*

Quality of the amplified products was checked by DL 2000 Marker (BEIJING TIANGEN Biotech, Co., Ltd.) before being sent to Sino Geno Max (Beijing) for further purification and then sequencing. Raw sequences were manually checked and then aligned using the reference 16s rDNA sequences (accession number: KC768792.1 for *V. parahaemolyticus* ATCC 17802; JX009143.1 for *V. fluvialis* NCTC 11327; EU204961.1 for *V. furnissii* M1; X76333.1 for *V. vulnificus* ATCC 27562T; DQ068934.1 for *V. mimicus* UN 11607; JX290082.1 for *V. cholera* E153) by Sequence Analysis Tool in RDP Release 11^1^.

#### Detecting *V. alginolyticus* in Infected Mice with Lamp

BALB/c female mice (20–23 g in weight) used in this study were purchased from the Academy of Military Medical Sciences (Beijing, China), and bred under specific-pathogen-free (SPF) conditions (Sakuma et al., 2013). All animal procedures complied with the institutional and national guidelines prescribed by the International Council for Laboratory Animal Science (ICLAS) the ministry of health of the People’s Republic of China. The mouse experiment was approved and supervised by Beijing Institute of Radiation Medicine Experiment Committee (2012-0131). The way of preparation of infected mouse model followed the methods described by Khajanchi et al. (2011), Angkasekwinai et al. (2014), and Liu et al. (2014, 2015).

The mice for virulence testing were divided into six groups (*G* = 6, *n* = 4); the bacterial strains injected include *V. alginolyticus* 17749 (G1), *V. fluvialis* 1A10009 (G2), *V. parahaemolyticus* 1A10122 (G3), *V. fischeri* 1H00021 (G4), *V. vulnificus* 1H00066 (G5) and non-*Vibrio* infected mice as the control group (G6). Mice were intraperitoneally injected with the *Vibrio* species above (1 × 10^6^ CFU/mL) resuspended in 1 × phosphate buffer saline (PBS), while G6 was injected with 1× PBS. After the injection, each BALB/c mouse was fed with sterile water only. Sampling from the infected and control groups were carried out 16 h post-infection by collecting blood. The blood obtained from eyeballs was blended with acid citrate dextrose solution (1.32% M/V sodium citrate, 0.48% M/V citric acid, and 1.47% M/V glucose) as the anticoagulant, and then were prepared for template DNA to run LAMP assay.

#### Detecting *V. alginolyticus* in Scallop Samples with LAMP

To simulate the detection of the pathogens in natural samples (i.e., seafoods), artificially *V. alginolyticus*-infected scallop samples were developed. Sterile scallop alkaline peptone water culture solutions (samples 4–6) were prepared and then added with *V. alginolyticus* in gradient. Final bacterial concentrations in solutions were 10^5^ CFU/mL (sample 1), 10^4^ CFU/mL (sample 2), and 10^3^CFU/mL (sample 3). These were then used to assay for the efficiency and sensitivity of the primers by real-time fluorescence LAMP kit method. Positive (bacterial culture) and negative (ddH_2_O) controls were used.

## Results

### Sensitivity of the LAMP Assay

**FIGURE 1:**
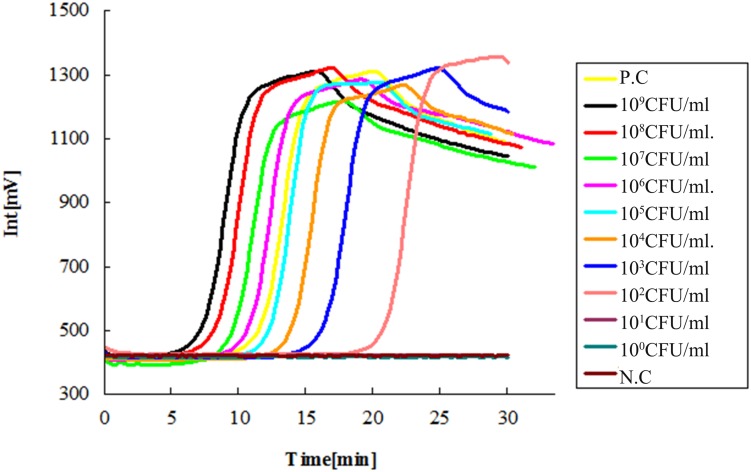
**Sensitivity of the LAMP assay with ESE-Quant tube scanner.** Suspensions of *Vibrio alginolyticus* ATCC 17749 were 10-fold serially diluted (1 × 10^0^ ∼ 1 × 10^9^ CFU/mL) and detected with the LAMP method using an ESE-Quant tube scanner. No amplification was detected in the 10-fold serial dilution equivalent to 1 × 10^0^ and 1 × 10^1^ CFU/mL DNA templates of *V. alginolyticus* standard strain ATCC 17749. *N.C as negative control, P.C as positive control.*

**FIGURE 2:**
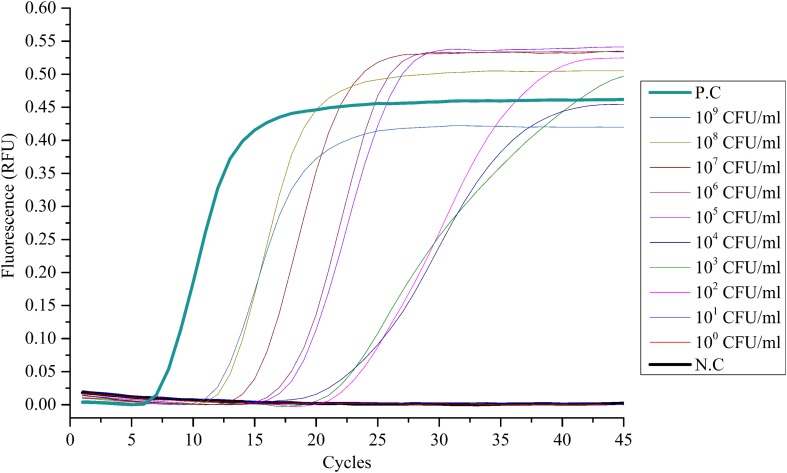
**Sensitivity of the LAMP assay with qPCR instrument.** Suspensions of *V. alginolyticus* ATCC 17749 were 10-fold serially diluted (1 × 100 ∼ 1 × 10^9^ CFU/mL) and detected with the LAMP method using a qPCR instrument. Only the suspensions of *V. alginolyticus* ATCC 17749 of 1 × 10^2^ ∼ 1 × 10^9^ CFU/mL could be detected. *N.C as negative control, P.C as positive control.*

The limit of detection (LOD) of the LAMP method was tested by serial dilutions, using ESE-Quant tube scanner and qPCR as the detecting device. The 10-fold serial dilution of *V. alginolyticus* ATCC 17749 with cells ranged from 1 × 10^0^ to 1 × 10^9^ CFU/mL as determined by plate counts. Results showed that no bacteria were detected at 1 × 10^0^ and 1 × 10^1^ CFU/mL DNA templates (**Figures 1** and **2**), suggesting that the LOD of the LAMP method using ESE-Quant tube scanner and qPCR device in this experiment were both 1 × 10^2^ CFU/mL.

### Specificity of the LAMP Assay

**FIGURE 3:**
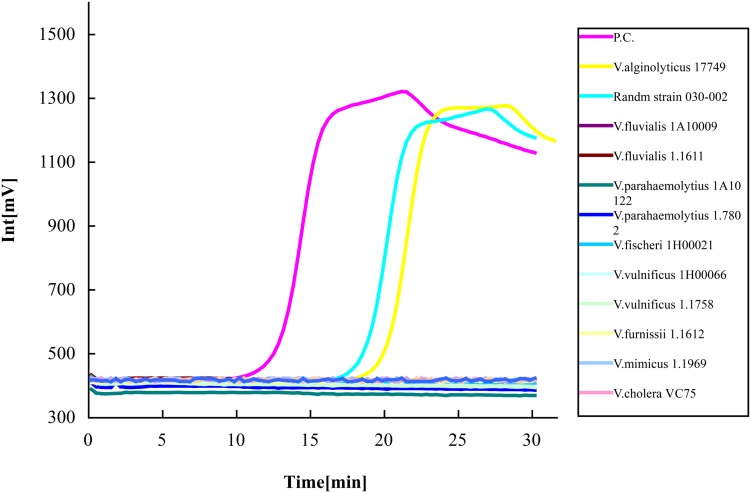
**Specificity of the LAMP-ESE-Quant Tube Scanner assay.** Eight suspensions of different *Vibrio* species (12 strains) adjusted to 10^6^ CFU/mL were detected with real-time fluorescence loop-mediated isothermal amplification kit using an ESE-Quant tube scanner. Only *V. alginolyticus* 17749 and strain 030-002 showed amplification curves out of the xx total number of strains tested. *N.C as negative control, P.C as positive control.*

The specificity of the LAMP method using two devices was observed. Altogether, eight different kinds of *Vibrio* species (12 strains) were used for detection, including two strains of *V. alginolyticus*, two strains of *V. fluvialis*, two strains of *V. parahaemolyticus*, two strains of *V. vulnificus*, and *V. fischeri*, *V. furnissii*, *V. mimicus*, *V. cholera* each of one strain. *Vibrio* strain 030-002 was randomly selected from the 92 strains of *V. alginolyticus*. Plasmid DNA containing the target gene isolated from *V. alginolyticus* 17749 was used as the positive control. Sterile ddH_2_O was used as the negative control. Using the LAMP-ESE-Quant Tube Scanner, aside from the positive control, the only successful amplified results were *V. alginolyticus* 17749 and the randomly chosen 030-002 strain (**Figure 3**). Fluorescence voltage increased to 550 mV after 26 min of the reaction which clearly separated the results.

**FIGURE 4:**
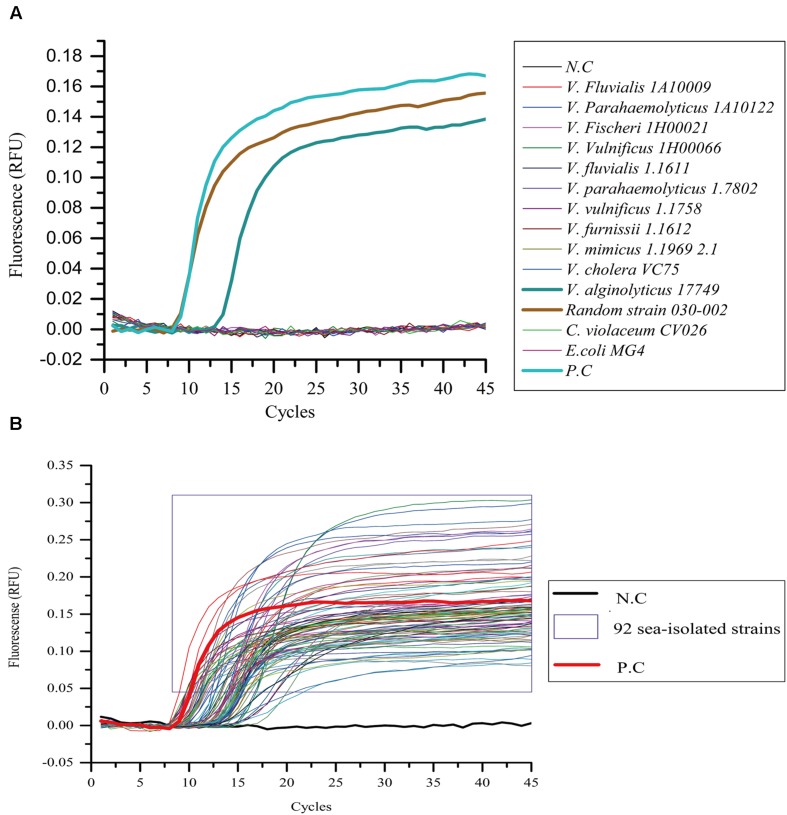
**Specificity of the LAMP-qPCR device assay.** All bacterial suspensions were adjusted to 10^6^ CFU/mL and detected with real-time fluorescence loop-mediated isothermal amplification kit method using a qPCR device. **(A)** Out of all the strains tested, only *V. alginolyticus* 17749 and strain 030-002 showed amplification. **(B)** All the 92 strains showed “S-shaped” amplification curves. *N.C as negative control, P.C as positive control*.

On the other hand, 14 different bacterial strains and 92 marine-isolated *V. alginolyticus* strains were detected by the LAMP-qPCR device. Among the 14 strains, an *E. coli* MG4 strain and a *C. violaceum* CV026 strain were added in addition to the 12 *Vibrio* strains used in **Figure 3**. The positive and negative controls were the same as described above. The results showed only two strains were positively detected, including *V. alginolyticus* 17749 and the randomly chosen 030-002 strain. The relative fluorescence units (RFU) rose to 0.0096 at the 14th cycle on strain 17749 and 0.0104 at the 10th cycle on the strain 030-002. They later stabilized at 0.10–0.13 from the 20th and 14th cycles, respectively toward the end of the reaction (**Figure 4A**). Similarly, all of the 92 marine-isolated *V. alginolyticus* strains tested using LAMP-qPCR device obtained “S” amplified curves in the RT-PCR assay. The intensity of the RFU varied from 0.82 to 0.30 (**Figure 4B**).

### Bacteria Verification by 16s rDNA Sequencing

The 16s rDNA results showed that the sequence homologies of 105 bacterial strains aligned with *V. alginolyticus* ATCC 17749, *V. fluvialis* NCTC 11327, *V. parahaemolyticus* ATCC 17802, *V. vulnificus* ATCC 27562, *V. furnissii* M1, *V. mimicus* UN 11607, and *V. cholera* E153 were in the range of 99.6–99.9%.

### Detection of the Infected Blood Samples

**FIGURE 5:**
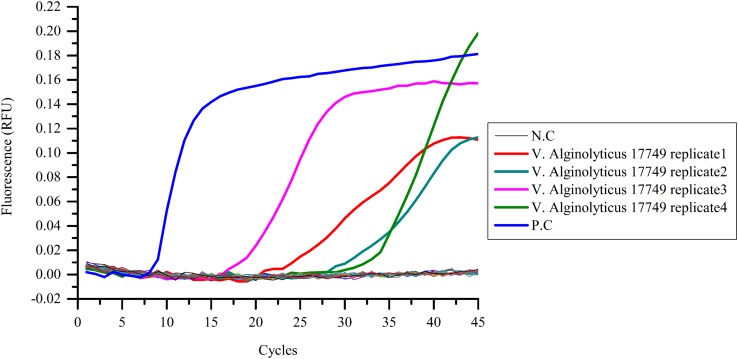
**Pathogenic bacterial detection in infected blood samples.** Blood samples were collected from *Vibrio* infected and uninfected mice, and template DNA was prepared for the LAMP assay. Among all of the chosen bacteria, amplification curves showed only in four replicates of *V. alginolyticus* 17749. *N.C as negative control, P.C as positive control.*

Real-time PCR successfully detected the presence of pathogens in the four replicates of blood infected with *V. alginolyticus* 17749 (G1), as evident in the obtained “S” curves (**Figure 5**). Cycle thresholds varied accordingly when the relative fluorescence values were raised to a separable range from the base line. The stable fluorescence values also differed, ranging approximately from 0.11 to 0.19.

### Detection of the Infected Scallop Alkaline Peptone Water Samples

The sterile and *V. alginolyticus* contaminated scallop alkaline peptone water samples assay results are shown in **Table 3**. The artificially contaminated samples showed positive amplification and that the results corresponded well to the experimental design, which shows that the detection method is stable and reliable.

**Table 3:** Summary of the detection of pathogens in the infected scallop alkaline peptone water.

Sample number	Samples containing *V. alginolyticus* amount (CFU/mL)	Test results
1	10^5^	Positive
2	10^4^	Positive
3	10^3^	Positive
4	0	Negative
5	0	Negative
6	0	Negative


*Positive and negative control test results are consistent. Artificially polluted samples 1∼3 were also successfully amplified. Test results correspond to the experimental design, which shows that the detection method is stable and reliable.*

## Discussion

### LAMP versus Conventional Methods

*Vibrio alginolyticus* as one of the most common species of aquatic bacteria and can thrive in oligotrophic conditions. However, once conditions become favorable (e.g., during infection or contamination of host), they exhibit rapid proliferation and growth causing food poisoning, which can easily induce diarrhea upon contact or ingestion (Caccamese and Rastegar, 1999; Chien et al., 2002). Thus, sensitivity and specificity of detection methods or assays are important in devising strategies to identify the presence of bacteria in pure cultures and especially in contaminated samples.

The conventional way of identifying *V. alginolyticus* is based on 16s rDNA identification accompanied by biochemical characterization. With the increase in the 16s rDNA sequences deposited in reference database, bacterial identification became easier using PCR amplification and sequencing. Phylogenetic and sequence analyses confirmed that 16s rDNA is a reliable indicator of species differentiation with the presence of polymorphic sites within particular organisms (Moreno et al., 2002). Meanwhile, biochemical characterization is based on different bacterial morphology and chemical reaction assays, which also provide a precise detection method. Although widely accepted, these two methods are both logistically limited (e.g., need for sophisticated equipment and facilities), and are very time-consuming (i.e., 3–10 days).

At present, significant improvements in technology for testing and detecting have been performed to help cure, mitigate and prevent the spread of such infections and contaminants. For example, improved PCR-assays using highly specificity primers have been gaining popularity as the accuracy is higher than conventional methods. The advent of molecular approaches such as specific PCR assay (Bej et al., 1999) and DNA probe hybridization (Gooch et al., 2001) paved the way for the development of rapid and easier methods of detection, which increased the efficiency of bacterial identification. Despite such improvements, use of PCR itself is still logistically limited to laboratories and impractical in field conditions and remote areas. Therefore, these molecular identification methods are still time-consuming (2–3 days) and none were appropriate for on field or on the spot monitoring.

The *toxR* gene was first found in *V. cholera* and generally exists only in *Vibrio* species, encoding a transmembrane transcriptional activator protein and mainly regulating the expression of virulence genes (Miller et al., 1987; Chakraborty et al., 2006). The sequence of a membrane tethering region in the *toxR* gene shows wide differences among *Vibrio* species, which makes it an excellent molecular target for the identification of *Vibrio* species (Carlos et al., 2000; Conejero and Hedreyda, 2003). Therefore, the gene polymorphisms in *toxR* in *Vibrio* species are greater than that in 16S rDNA.

In this study, based on LAMP technology, a set of six specific primers targeting the *toxR* gene sequence of *V. alginolyticus* were designed and tested, which could specifically identify eight independent regions of the targeted gene. Along with *Bst* WarmStart^TM^ DNA polymerase that had strand substitution activity, strand substitution reaction was successfully carried out along with the synthesis of complementary strand on the target DNA region. Amplification reaction was accomplished at 60–65°C, and the entire detection process was completed within 20–60 min, which was significantly faster compared to previous techniques.

At the same time, using the primers we designed coupled with the RT-PCR and ESE-Quant Tube Scanner, high sensitivity and 100% specificity of the rapid and simple LAMP assay was obtained. The sensitivity of the new method on the two detection platforms (ESE-Quant tube scanner and qPCR device) was highly consistent (**Figures 1** and **2**). Moreover, compared to conventional 16s rDNA PCR assays, the LAMP assay was more sensitive and the results were more intuitive. In pure cultures, the amplification curves generated via ESE-Quant Tube Scanner clearly showed the separation of *V. alginolyticus* and non-*V. alginolyticus* (**Figure 3**), and the same pattern was observed when qPCR was used (**Figure 4**). The above results were consistent with results of previous studies (Fall et al., 2008; Han and Ge, 2008; Yamazaki et al., 2008), and also provided data showing the application of this assay not only in field detection but also in routine research laboratories.

Previous investigations found that the LOD for *V. parahaemolyticus* was 1.1 × 10^5^ CFU/mL (Chen and Ge, 2010), 10^4^ CFU/mL for *Bacillus bacilliformis* (Angkasekwinai et al., 2014) and 2 CFU/25 g for *V. cholerae* in food and environmental samples (Garrido-Maestu et al., 2015). Targeting the *toxR* gene in our study, we showed that the LOD of *V. alginolyticus* pure culture could be as low as 10^2^ CFU/ml, which was consistent with previous results that detected *V. alginolyticus* by targeting the *gyrB* gene using a LAMP method (Cai et al., 2010). This result verifies the hypervariability and heterogeneity of the *toxR* gene (Chen and Ge, 2010) that can be used to distinguish *V. alginolyticus* strains. Compared to primers targeting *tlh/ldh* gene (Yi et al., 2014), our data also showed better accuracy, indicating precision of this rapid and simple LAMP assay.

### Detection *V. alginolyticus* in Mouse Blood and Scallop Samples with LAMP Assay

Previous studies performed in aquatic animal models have tested for the presence of *B. bacilliformis* in contained conditions. However, the test strain demonstrated limited capacity for growth when artificially fed to *L. longipalpis* (Angkasekwinai et al., 2014). Meanwhile, *Vibrio* has strong infectivity and pathogenicity (Oates et al., 2012; Xiong et al., 2014) making it a good model system to study. In fact, the intraperitoneal LD_50_ in mice reached 1 × 10^8^ to 1 × 10^9^ CFU of *V. alginolyticus* E0666 (Liu et al., 2014). However, as a relatively newly classified bacterium from *V. parahaemolyticus* in Buchanan and Gibbons (1974), only few studies testing *V. alginolyticus* in animal models and much less on mammalian models have been performed.

Our study performed excellently not only in pure bacterial cultures but also in infected mice and artificially contaminated samples. The mice injected with *V. alginolyticus* and other bacteria immediately exhibited constant chilling and shaking compared with the control group, which were defensive mechanisms against infection. Then, 16 h after injection, physiological states of the infected mice became worse, which varied in between groups. The immune system of the mice played an important role in metabolizing and phagocytosing some of the bacteria, resulting in an expected decrease in the concentration of the pathogens in the blood. In spite of such pathological processes, our LAMP assay still detected the existence of *V. alginolyticus* in the blood of the mice. To the best of our knowledge, this is the first time that *V. alginolyticus* has been detected directly from extracted mammalian blood. Using the newly described rapid and simple LAMP assay, we provided a more accurate method for the detection of *V. alginolyticus.*

Same as the detection in the model animal, *V. alginolyticus* in artificially contaminated scallop alkaline was also successfully detected. In addition, with the ESE Tube Scanner, our LAMP assay is applicable to rural communities, inaccessible locations and field use. To secure safety and sustainability of marine fisheries, detection and prevention of pathogenic diseases, timely warning and control of food safety problems are of great importance.

### Advantages of Improved LAMP Assay

Other than high specificity and sensitivity, time-saving and intuitive monitoring of results, the feasibility for the method to be used *en masse* in the future is highly dependent on availability of the materials such as the fluorochrome SYTO^®^-9 and ineral oil that could be easily obtained.

The use of two platforms (i.e., Real-Time PCR and ESE-Quant Tube scanner) both obtained 100% inclusion and exclusion. This means that the LAMP assay for *V. alginolyticus* that we described in this study was very stable and efficient under different environments. The ESE-Quant tube scanner system is the most commonly used LAMP detection platform, which is portable and doesn’t need the experiment operator to have a high level of professional knowledge. On the other hand, as real-time fluorescent quantitative PCR instruments have become common in most laboratories, the subsequent verification experiments were mainly detected on the qPCR platform, so as to provide evidence for its application in laboratories. Therefore our results showed that this method could be carried out both in laboratory and field conditions.

## Conclusion

Valued for its sensitivity, specificity, and rapidity in detecting pathogens in pure culture and artificially contaminated samples under different situations, our LAMP assay is a promising tool and which could be used by both professionals and non-professionals worldwide.

## Author Contributions

All authors were involved in design of the study and interpretation of data. All authors approved the final version. KF and JL helped conceive project, designed and performed experiments and wrote the manuscript. YW conceived project, designed, and interpreted experiments. JL executed experiments and wrote the manuscript. HY conceived and interpreted experiments. LS and LZ helped conceive project and edit the manuscript.

## Conflict of Interest Statement

The authors declare that the research was conducted in the absence of any commercial or financial relationships that could be construed as a potential conflict of interest.

## Footnotes

1 http://rdp.cme.msu.edu/index.jsp
